# Transcriptomic analysis of tomato carpel development reveals alterations in ethylene and gibberellin synthesis during *pat3/pat4 *parthenocarpic fruit set

**DOI:** 10.1186/1471-2229-9-67

**Published:** 2009-05-29

**Authors:** Laura Pascual, Jose M Blanca, Joaquin Cañizares, Fernado Nuez

**Affiliations:** 1 Instituto de Conservación y Mejora de la Agrodiversidad Valenciana (COMAV), Universidad Politécnica de Valencia, Camino de Vera s/n, 46022 Valencia, Spain

## Abstract

**Background:**

Tomato fruit set is a key process that has a great economic impact on crop production. We employed the Affymetrix GeneChip Tomato Genome Array to compare the transcriptome of a non-parthenocarpic line, UC82, with that of the parthenocarpic line RP75/59 (*pat3/pat4 *mutant). We analyzed the transcriptome under normal conditions as well as with forced parthenocarpic development in RP75/59, emasculating the flowers 2 days before anthesis. This analysis helps to understand the fruit set in tomato.

**Results:**

Differentially expressed genes were extracted with maSigPro, which is designed for the analysis of single and multiseries time course microarray experiments. 2842 genes showed changes throughout normal carpel development and fruit set. Most of them showed a change of expression at or after anthesis. The main differences between lines were concentrated at the anthesis stage. We found 758 genes differentially expressed in parthenocarpic fruit set. Among these genes we detected cell cycle-related genes that were still activated at anthesis in the parthenocarpic line, which shows the lack of arrest in the parthenocarpic line at anthesis. Key genes for the synthesis of gibberellins and ethylene, which were up-regulated in the parthenocarpic line were also detected.

**Conclusion:**

Comparisons between array experiments determined that anthesis was the most different stage and the key point at which most of the genes were modulated. In the parthenocarpic line, anthesis seemed to be a short transitional stage to fruit set. In this line, the high GAs contends leads to the development of a parthenocarpic fruit, and ethylene may mimic pollination signals, inducing auxin synthesis in the ovary and the development of a jelly fruit.

## Background

Fruit development and ripening are key processes for crop production, tomato has been widely used as a model for the regulation of these processes [1]. Tomato is a fleshy and climacteric crop that has several advantages as a fruit development model: economic importance as a crop, small genome, short generation time, availability of transformation protocols and genetic and genomic resources [2,3].

Fruit development can be divided into several phases [4]. The first one comprises the initiation of the floral primordia and carpel development up to anthesis. At this point, the development arrests and either of two paths can be taken: if it is pollinated and fertilized, the flower will resume the process, reaching fruit set; otherwise, the carpel will senesce. The second phase starts after fruit set and is characterized by fruit growth due to cell division. During the third phase, the fruit growth continues until the fruit reaches its final size, but this enlargement is mainly due to cell expansion. These growing phases are followed by ripening and senescence.

Fruit set is affected by multiple environmental conditions, such as light, humidity and temperature which must be within a certain range to allow fruits to develop. A better understanding of the developmental and environmental factors that control fruit set would lead to an optimization of growing conditions that might improve crop production.

Besides the influence of these external factors in the control of fruit set the existence of a hormonal control is also obvious and has been demonstrated by various studies reviewed by Ozga [5] and Srivastava [6]. In tomato, this process is independent of embryo development, and the linkage between the processes can be broken. Parthenocarpy, the production of fruits without seeds, is common in this species and can be caused by natural mutations, environmental factors or hormone treatments, reviewed by Gorguet [7]. Gibberellins (GAs) and auxins play a crucial role in this process in tomato, although it appears that other plant regulators might be involved. The role of these hormones has been demonstrated by the measuring of endogenous levels in pollinated ovaries, in the unpollinated ovaries of parthenocarpic lines and by exogenous application [8]. Several genes are also described as being involved in fruit set control: among others, *Aux/IAA *transcription factor *IAA9*. Plants with *IAA9 *inhibited present auxin related growth alterations as well as fruit development triggered before fertilization, giving rise to parthenocarpy [9]. Transgenic tomato plants with down-regulated expression of *TM29*, a tomato *SEPALLATA *homologue, develop parthenocarpic fruits and produce aberrant flowers with morphogenetic alterations in the organs of the inner three whorls [10]. Arabidopsis mutant *arf 8 *(auxin response factor 8) and tomato plants carrying *ARF8 *transgenic constructions also develop parthenocarpic fruits [11,12].

Although natural and artificial mutants have demonstrated the existence of a genetic control of fruit set, little is known about how it works. Parthenocarpic fruit development is a trait of great interest as it provides an ideal framework for studying the factors affecting fruit set in addition to improving fruit set in harsh conditions.

There are three main sources of parthenocarpic growth in tomato: *pat, pat-2 and pat3/pat4 *[13-15]. These lines are able to produce parthenocarpic fruits after emasculation that have nearly the same properties as fruits obtained after pollination and fertilization. The *pat *mutant has been widely analyzed, although it presents pleiotropic effects that affect not only fruit set but also flower morphology, with abnormal stamen and ovule development [16]. The *pat-2*, a single recessive gene with no pleiotropic effects, is responsible for the parthenocarpy in the "*Severianin*" cultivar [17]. The *pat-3/pat-4 *system (RP75/59) was described in a progeny from a cross between *Atom *× *Bubjekosko*. Studies of RP75/59 have finally led to the acceptance of a genetic model with two genes, *pat-3 *and *pat-4 *[18,19]. GAs content in the ovaries of these three mutants is altered even before pollination and seems to play a key role in the parthenocarpic phenotype [8,20,21]. Unfortunately, little more is known about these genetic systems; none of the genes have been cloned and only the *pat *gene has been mapped [22].

As of this work, no global analysis of gene expression during parthenocarpic fruit set has been published for tomato. Most of the studies related to this crop have been focused on later stages of fruit development and ripening [23-25], and only a couple of recent studies have analyzed the fruit set at a transcriptomic level [26,27]. In this work, the Affymetrix GeneChip Tomato Genome Array was used to study the developmental processes that occur during carpel development and fruit set. We employed a non-parthenocarpic line, UC82, and the facultative parthenocarpic line, RP75/59 (*pat3/pat4 *mutant), to identify the genes modulated throughout carpel development and fruit set and to determine the differences between parthenocarpic and normal fruit set. We have identified changes in cell division genes that imply cell cycle alterations in the parthenocarpic line. In addition, differences in several hormone-related genes are relevant and asses the importance of GAs for parthenocarpic development and a new role for ethylene in this process.

## Results

### Transcriptomic analysis of tomato carpel development and fruit set

Carpel development in tomato arrests at anthesis and is not resumed until pollination and successful fertilization. However, the facultative parthenocarpic line RP75/59 sets fruits in absence of pollination.

To study carpel development, fruit set and parthenocarpic development, we compared the non-parthenocarpic UC82 and RP75/59 transcriptomes. UC82 was selected as the normal development control due to its high percentage of fruit set, which is higher than 90%, and its phenotypic resemblance to RP75/59. In order to analyze the carpel development and fruit set of both lines, flowers were collected at four time points: flower bud, flower bud to pre-anthesis, anthesis and 3DPA (days post anthesis).

The expression of *PCNA *(proliferation cell nuclear antigen), a cell division marker, was tested by quantitative PCR (QPCR) to monitor the developmental arrest at anthesis and the restart that takes place when fruit sets (Table 1). In UC-82, *PCNA *expression decreases at anthesis and at 3DPA increases. In RP75/59, the pattern was similar, although the expression at anthesis was higher.

**Table 1:** Differentially expressed genes in the parthenocarpic development tested by QPCR.

Array probe set	Gen description	Assigned SGN	Ant_E QPCR	3DPA_E QPCR	Ant_E Array	3DPA_E Array
Les.4978.1.S1_at	DNA replication licensing factor		0.53	-0.53	1.13	-0.29

Les.5343.1.S1_at	Cell division control protein 6	SGN-U323296	0.24	-0.22	1.23	-0.44

Les.3520.1.S1_at	Cyclin d3-2	SGN-U321308	1.36	-0.64	1.38	-0.44

Les.5917.1.S1_at	ACC oxidase ACO5 (synthesis-degradation)	SGN-U323861	1.8	0.66	1.76	0.31

LesAffx.67531.1.S1_at	AXR2| IAA7 (response)		0.77	-1.91	0.72	-2.59

Les.3707.1.A1_at	Auxin-responsive protein IAA2 (response)	SGN-U339965	-1.6	-2.1	0.16	-2.15

Les.63.1.S1_at	GA20-oxidase 3 (synthesis-degradation)	SGN-U321270	2.41	1.08	3.65	1.49

Les.65.1.S1_at	GA20-oxidase 2 (synthesis-degradation)	SGN-U333339	-0.08	-0.89	0.38	-1.16

Les.2949.1.S1_at	PCNA		0.93	-0.09	1.53	-0.42

Ant_E and 3DPA_E columns showed the fold change for each gene in RP75/59 with respect to UC-82, according to the QPCR and to the microarray, after log2 transformation.

**Figure 1:**
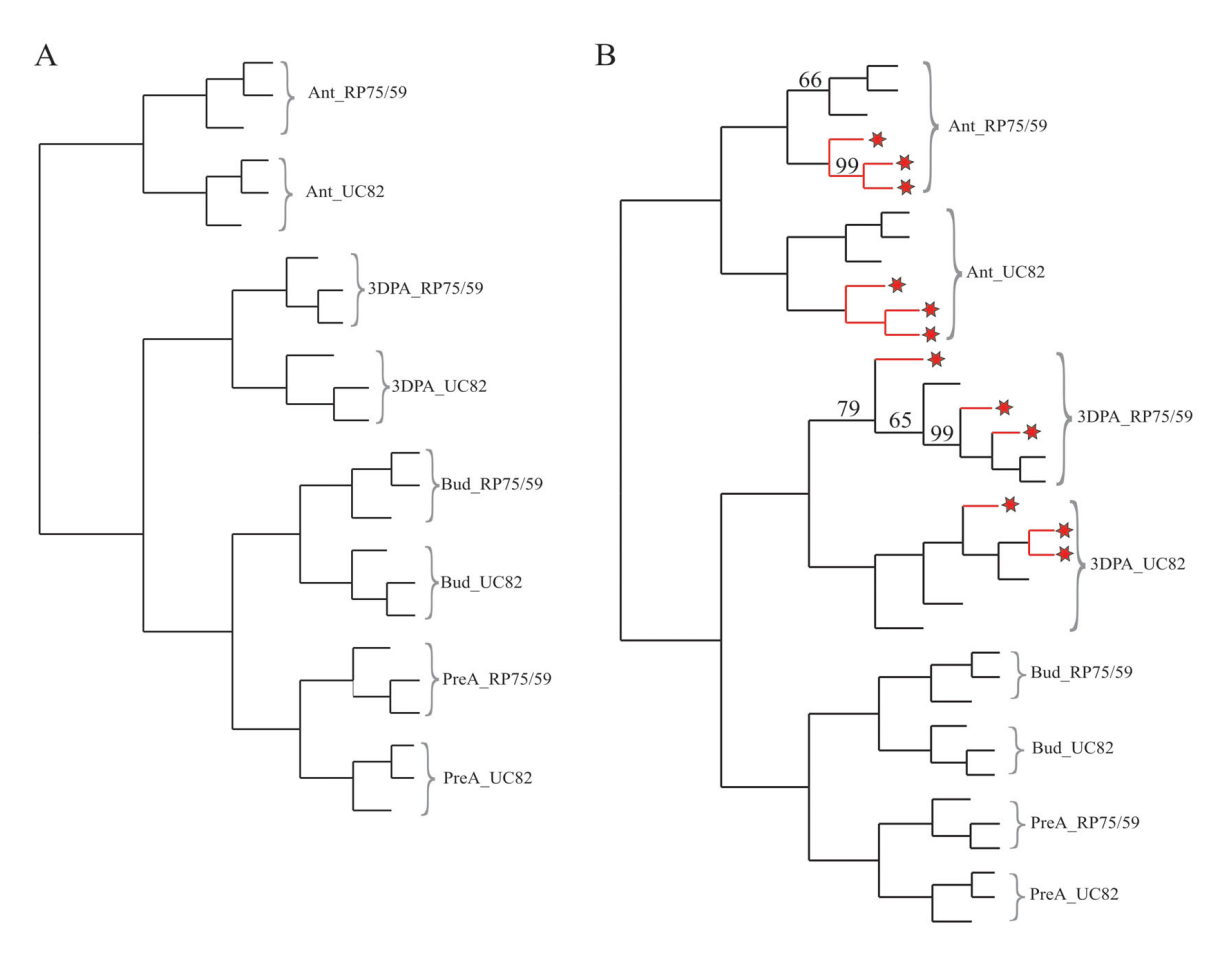
**Samples Cluster**. Samples clustered by UPGMA with bootstrap according to the differentially modulated genes. Bud (petal length between 4.5 and 7 mm), Bud_Preant (petal length between 7.5 and 9 mm), Ant (anthesis), Ant_E (anthesis emasculated prior to anthesis), 3DPA (3 days after anthesis) and 3DPA_E (3DPA emasculated prior to anthesis). Bootstrap values are only shown when lower than 100. **A**. Cluster of the non-emasculated samples. **B**. Cluster of all stages and conditions. * Samples emasculated before anthesis.

Three biological replicates of each line and stage were hybridized with the GeneChip Tomato Genome Array (Affymetrix). To analyze the different stages of development, we discarded the constant genes in order to avoid background noise and clustered the samples according to gene expression by UPGMA.(Figure 1A). Replicates from the same line and stage were clustered together in all cases. Flower bud stages and flower bud to pre-anthesis stages were grouped together and were closer to 3DPA stages than were anthesis samples.

### Differentially expressed genes throughout carpel development and fruit set

**Figure 2:**
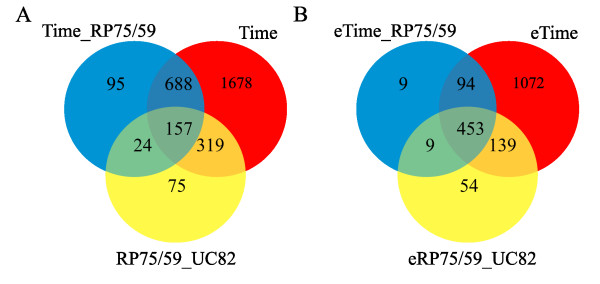
**Venn diagram**. **A**. The number of genes in the Tomato Affymetrix GeneChip that changed in TIME (during UC-82 carpel development), TIME_RP75/59 (genes that changed throughout RP75/59 development, but in a different way than in UC-82) and RP75/59_UC82 (genes whose expression was different between the two lines, regardeless of whether they changed over time). **B**. The number of genes in the Tomato Affymetrix GeneChip that changed in emasculated stages, eTIME (changed between anthesis and 3DPA in UC-82), eTIME RP75/59 (changed in a different way between anthesis and 3DPA in RP75/59) and eUC-82vsRP75/59 (genes whose expression was different between the two lines).

To identify processes altered in parthenocarpic carpel development in tomato, we compared the transcriptome of the non-partenocarpic UC-82 line with that of the partenocarpic RP75/59 line. Differentially expressed genes were extracted with maSigPro [28], which is designed for the analysis of single and multiseries time course microarray experiments. The method first defined a general model for the data according to the experimental variables and their interactions, then extracted those genes that were significantly different from the model. Secondly, a selection procedure was applied to find the significant variables for each gene. The variables defined in our analysis were: TIME (for those genes that changed during UC-82 carpel development), TIME RP75/59 (for those genes that changed during RP75/59 development, but in a different way than in UC-82) and UC-82vsRP75/59 (for those genes whose expression was different between the two lines, regardless of whether they changed over time) (Figure 2A).

**Figure 3:**
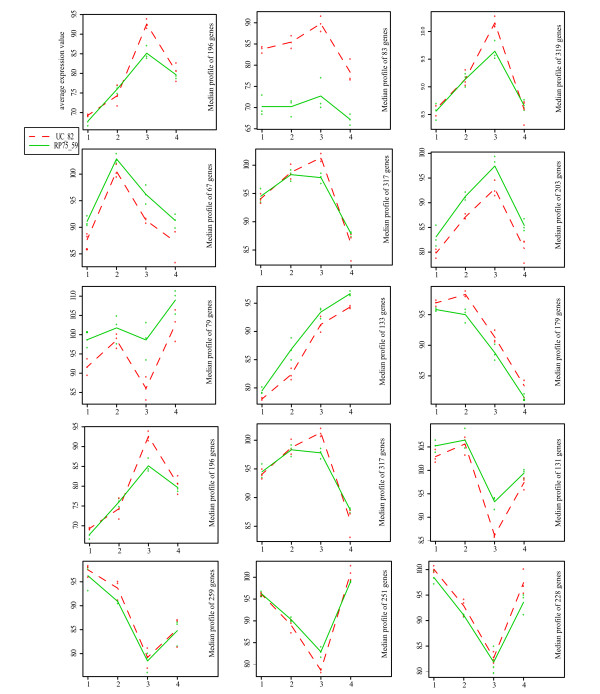
**Clustering of genes that changed during normal carpel development and fruit set (TIME)**. Cluster analysis of genes differentially expressed during UC-82 carpel development; genes clustered by their expression in UC82 and RP75/59; the expression patterns of the two lines represented separately. Level of expression in the Y axis. Stages of development in the X axis 1, 2, 3 and 4 are, flower bud, from bud to pre-anthesis, anthesis and 3DPA respectively.

2842 differentially expressed genes were associated to the TIME variable (Additional file 1). The expression patterns corresponding to those genes were grouped in 15 clusters (Figure 3). Most of the differentially expressed genes showed a change of expression at or after anthesis. Between the two lines, the clusters with the greatest differences were the ones with different levels of expression throughout entire development, and the ones where the differences between lines were concentrated at anthesis.

RP75/59 is a strongly facultative parthenocarpic tomato line. Even when the flowers are not emasculated it can set parthenocarpic fruits. We selected 1358 differentially expressed genes in RP75/59 (variables TIME RP75/59 and UC-82vsRP75/59) (Additional file 2). Most of these genes also changed in UC82 during TIME (Figure 2A).

**Figure 4:**
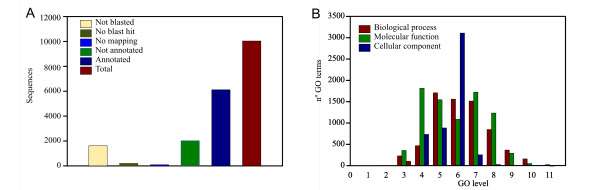
**Array annotation summary**. **A**. Annotation process results for Tomato Affymetrix GeneChip. **B**. GO level distribution chart for Tomato Affymetrix GeneChip.

To identify which biological processes are involved in carpel development and fruit set, we analyzed the Gene Ontology terms (GO terms) of the differentially expressed genes. Even though Affymetrix provides an annotation of the arrays, we found it incomplete as only a thousand probes had GO terms assigned. To improve the functional analysis of the genes, we re-annotated the array using the blast2GO package [29](Additional file 3). At the end, 6121 probe sets were annotated (Figure 4A). The annotated GO terms ranked from level 2 to level 11, but were concentrated around level 6 (Figure 4B).

Using the FatiGO program [30] we extracted the terms that were over- or underrepresented in the differentially expressed genes associated with the variable TIME with respect to the rest of the array (Table 2). In our set of genes, regulation of cell cycle and regulation of progression through cell cycle, were over-represented. In addition, we found that RNA splicing, RNA metabolic process, RNA processing, biopolymer metabolic process, biopolymer catabolic process, macromolecule metabolic process and vesicle-mediated transport were underrepresented in our set of genes.

**Table 2:** Significantly different GO terms in normal development

GO term	Level	Percentage TIME	Percentage Array	Adj. pvalue
Biopolymer metabolic process	4	18	27.06	9.51E-007

mRNA metabolic process	6	0	2.18	3.20E-005

mRNA processing	7	0	2.43	8.05E-004

RNA metabolic process	5	4.52	8.94	1.70E-003

RNA splicing	7	0.14	2.61	1.70E-003

Macromolecule metabolic process	3	41.26	48.71	2.92E-003

Biopolymer catabolic process	5	1.04	3.3	1.17E-002

RNA splicing, via transesterification reactions with bulged adenosine as nucleophile	9	0	4.89	1.94E-002

RNA splicing, via transesterification reactions	8	0	2.51	2.03E-002

Regulation of cell cycle	5	1.98	0.52	3.26E-002

Regulation of progression through cell cycle	6	2.14	0.57	3.26E-002

RNA processing	6	1.53	3.84	4.31E-002

Vesicle-mediated transport	5	0.85	2.7	4.59E-002

GO Terms that were over- or under- represented in the genes modulated during normal carpel development and fruit set (TIME), with respect to the rest of the array.

**Figure 5:**
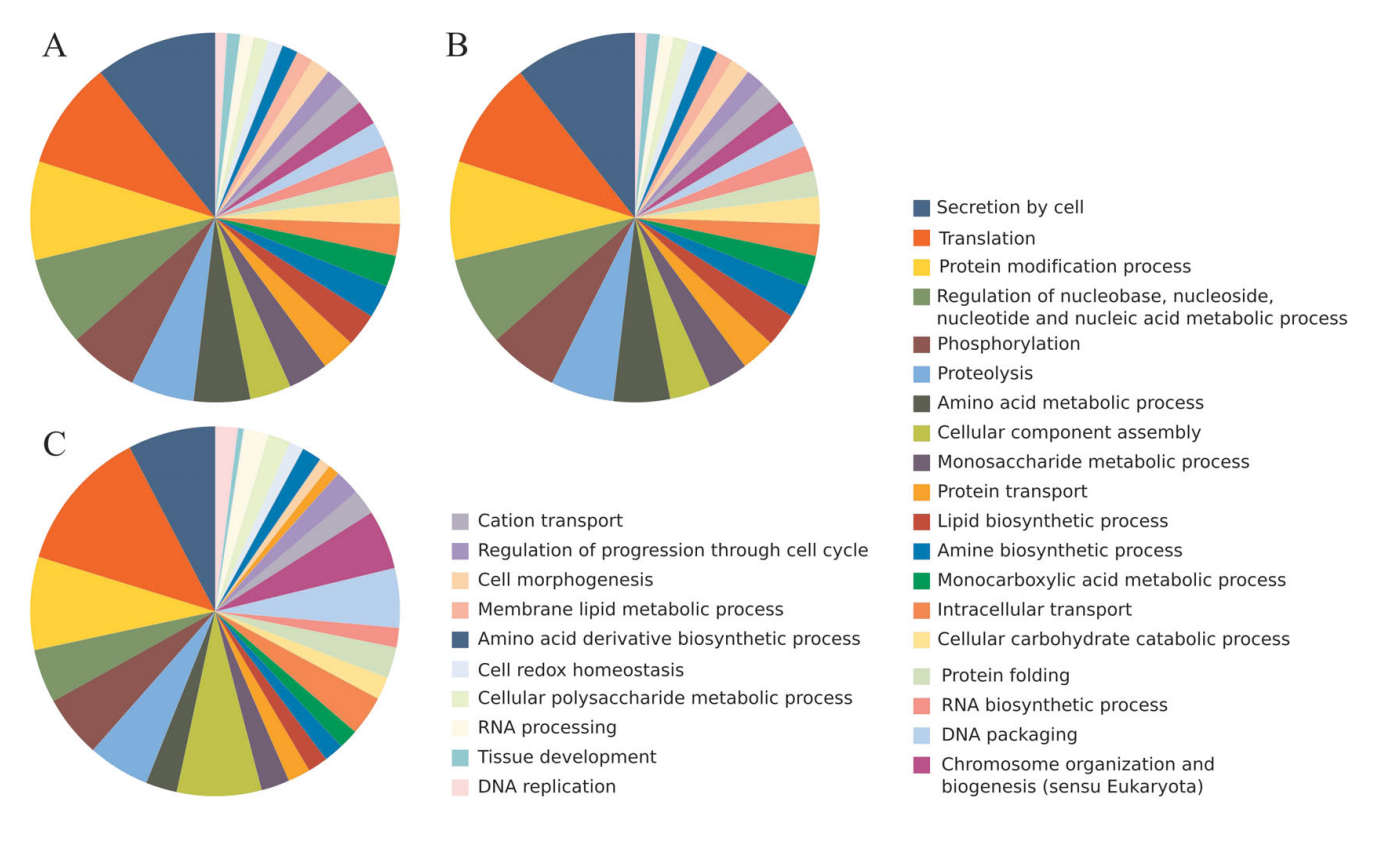
**Distribution of GO terms of the differentially expressed genes**. Frequencies of the GO terms in the differentially expressed genes. **A**. During UC-82 carpel development (TIME). **B**. In the differentially expressed genes in RP75/59 with respect to UC-82(TIME_RP75/59). **C**. In the parthenocarpic fruit set with respect to normal fruit set. eTIME RP75/59 and eUC-82vsRP75/59 (genes that changed in a different way in RP75/59 from than in UC-82 between anthesis emasculated and 3DPA emasculated and genes whose expression level was different between the two lines at this stages).

To identify other processes that may be involved in fruit set, we analized the GO terms whose frequency was greater than 2%. In the TIME differentially expressed genes (Figure 5A), we found genes related to metabolism, protein metabolism, secretion by cell, phosphorylation, monosaccharide metabolism as well as genes related to cell cycle and DNA synthesis, such as regulation of nucleobase, nucleoside, nucleotide and nucleic acid metabolic process, chromosome organization and biogenesis (sensu Eukaryota), DNA packaging, regulation of progression through cell cycle and cell morphogenesis. We also checked the GO terms of the differentially expressed genes in RP75/59 (variables TIME RP75/59 and UC-82vsRP75/59) (Figure 5B). With respect to the terms of the variable TIME, we found four new terms present more than 2%: membrane lipid metabolic process, DNA replication, cell redox homeostasis and tissue development. The rest of the terms were also present in the variable TIME with similar percentages.

### Differentially expressed genes in parthenocarpic fruit set

As RP75/59 can produce both seeded and seedless fruits. To improve the differential analysis, we forced parthenocarpic development in RP75/59 by emasculating the flowers 2 days before the anthesis to prevent natural pollination. Only UC82 flowers, and not RP75/59 flowers were pollinated at anthesis. The transcriptomes of the emasculated and non-emasculated flowers were quite similar (Figure 1B). We focused our analysis on anthesis and 3DPA, where the differences between lines were greater, comparing the transcriptomes of the two lines under these conditions.

We detected the genes whose expression changed between emasculated anthesis and emasculated 3DPA. Three new variables were defined for the emasculated stages: eTIME (for those genes that changed between anthesis and 3DPA in UC-82), eTIME RP75/59 (for those genes that changed between anthesis and 3DPA in a different way in RP75/59 from that in UC-82) and eUC-82vsRP75/59 (for those genes whose expression was different between the two lines) (Figure 2B). We selected 758 genes differentially expressed (Additional file 4), the ones assigned to eTIME RP75/59 and eUC-82vsRP75/59, those that were differentially expressed between parthenocarpic and normal fruit set.

**Figure 6:**
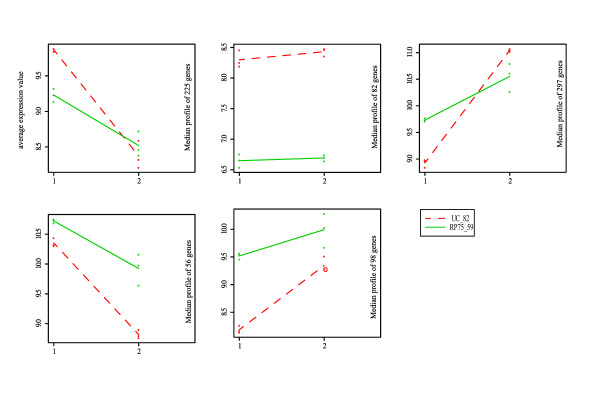
**Clustering of genes that changed during parthenocarpic fruit set**. Cluster analysis of genes differentially expressed in parthenocarpic fruit set with respect to normal fruit set (eTIME RP75/59 and eUC-82vsRP75/59) genes clustered by their expression in UC82 and RP75/59, expression pattern of two lines represented separately. Level of expression in the Y axis. Stages of development in the X axis 1 and 2 are, e anthesis and e 3DPA respectively.

To explore the expression changes, we grouped these genes into 5 clusters (Figure 6). There were two groups of genes that had a higher expression in RP75/59 at anthesis and 3DPA, one that had a higher expression in UC-82 at both stages, one where the expression was higher in UC-82 at anthesis and one where the expression was higher in RP75/59 at anthesis but lower at 3DPA.

To identify the biological processes involved in parthenocarpic fruit set, we analyzed the GO terms that label the differentially expressed genes. We found mainly the same terms as in the analysis of the TIME variable and three new terms: DNA replication (which was present in TIME RP75/59 and RP75/59vsUC82), RNA processing and amino acid derivate biosynthetic process (Figure 5C).

We also extracted the GO terms that were over- or underrepresented in the differentially expressed genes associated with the variables eTIME RP75/59 and eRP75/59vsUC82 with respect to the rest of the array using the Fatigo program (Table 3). We found that many processes related to chromatin organization were overrepresented, such as chromatin assembly, protein-DNA complex assembly, chromosome organization and biogenesis and DNA packaging, which might be related to differences in cell division. Nucleoside diphosphate metabolic process and macromolecular complex assembly were also overrepresented.

**Table 3:** Significantly different GO terms in parthenocarpic fruit set

GO term	Level	Percentage eTIME RP75/59 eRP75/59vsUC82	Percentage Array	Adj. pvalue
Chromatin assembly	9	24.1	4.67	1.71E-005

Organelle organization and biogenesis	4	14.1	5.75	1.71E-005

Chromatin assembly or disassembly	8	13.89	2.66	1.71E-005

Establishment and/or maintenance of chromatin architecture	7	10.45	2.24	3.58E-005

Protein-DNA complex assembly	8	13.19	2.99	2.43E-004

Chromosome organization and biogenesis (sensu Eukaryota)	6	6.8	1.68	2.70E-004

Chromosome organization and biogenesis	5	6.34	1.56	2.70E-004

DNA packaging	6	6.8	1.68	2.70E-004

Macromolecular complex assembly	7	14.93	5.89	2.65E-003

Nucleoside diphosphate metabolic process	6	1.29	0	1.54E-002

GO Terms that were over- or underrepresented in the genes modulated differentially in parthenocarpic fruit set (eTIME RP75/59 and eRP75/59vsUC82) with respect to the rest of the array.

### Microarray validation

**Figure 7:**
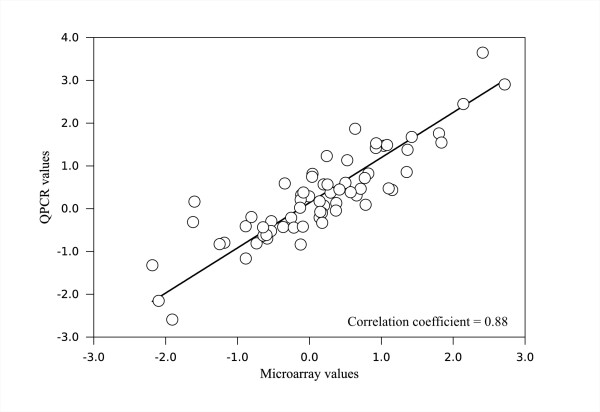
**Microarray validation**. Correlation between the microarray data and the QPCR results. X axis, fold change between RP75/59 and UC-82 in the microarray data. Y axis, fold change according to the QPCR results, data has been log2 transformed to made them comparable with the microarray results.

Array results were validated by QPCR, PCNA and 10 genes out of the differentially expressed along carpel development (TIME) were tested in the 6 stages analyzed (bud, bud to pre-anthesis, anthesis, emasculated anthesis, 3DPA and emasculated 3DPA). In the QPCR we used actin gene as reference, the fold change between RP75/59 and UC-82 was calculated and the result was log 2 transformed to made the data comparable with the microarray. In spite of the differences between both methods, the correlation was 0.88 (Figure 7). The fold change between RP75/59 and UC-82 of 9 genes that were also differentially expressed between the parthenocarpic and no-parthenocarpic lines are shown in table 1.

### Expression of cell division and cycle genes

As was demonstrated by the GO term analysis, the cell cycle related genes were modulated during carpel development and normal fruit set (variable TIME), which maybe caused by the cell cycle stop that takes place at anthesis. Additional file 5 shows all of the cell cycle and cell division genes that changed throughout carpel development and fruit set. There were two main groups of genes, differentiated by their expression patterns. Group 1 genes were genes whose expression was higher at flower bud, decreased when approaching anthesis, and increased at 3DPA, signifying, higher expression at the higher cell division stages. All the cyclins and cyclin-dependent kinases were placed in this group except for one a cyclin H homologue. Group 2 genes consisted of genes with higher expression at pre-anthesis and anthesis, when cell duplication is lower.

In order to evaluate the differences in cell cycle that maybe caused by parthenocarpic development, we also checked differentially modulated genes in parthenocarpic fruit set with respect to normal fruit set (variables eTIME RP75/59 and eRP75/59vsUC82) (Table 4). All of these genes were also differentially expressed during TIME (group 1). In UC82 (normal fruit set), they had a higher expression at the 3DPA stage and a lower expression at anthesis. In RP75/59 (parthenocarpic fruit set), these genes were more activated at anthesis, and so the activation at 3DPA was slighter than in UC82.

**Table 4:** Cell division and cycle genes

Array probe set	Gen description	Assigned SGN	RP75/59 Ant_E	UC82 Ant_E	RP75/59 3DPA_E	UC82 3DPA_E
Cell division related						

Les.2949.1.S1_at	PCNA2 proliferating cell nuclear antigen 2 (PCNA2)	SGN-U318069	11.39	9.86	11.98	12.4

LesAffx.67274.1.S1_at	Aurora kinase b		7.04	6.36	7.72	8.29

Cell cycle arrest						

Les.450.1.S1_at	Cyclin-dependent kinase inhibitor 3	SGN-U322716	7.78	7.82	8.57	9.34

DNA replication licensing factor						

Les.4978.1.S1_at	DNA replication licensing factor	SGN-U322656	10.42	9.29	10.89	11.18

Les.5283.1.S1_at	DNA replication licensing factor mcm5		10.54	9.18	10.91	11.2

G2/M transition of mitotic cell cycle						

Les.3713.1.S1_at	Cyclin-dependent kinase		8.36	7.1	10.47	11.32

M phase of meiotic cell cycle						

Les.89.1.S1_at	RAD51	SGN-U330095	7.44	6.77	8.37	8.98

M phase of mitotic cell cycle						

LesAffx.25483.1.S1_at	Anaphase-promoting complex subunit 11 homolog		9.63	8.93	9.75	10.12

LesAffx.60610.2.S1_at	Ubiquitin-conjugating enzyme E2C		8.63	7.49	11.23	12.24

Regulation of progression through cell cycle						

Les.107.1.S1_at	Cyclin a2	SGN-U326225	6.14	5.54	7.77	8.54

Les.351.1.S1_at	Cyclin-dependent kinase	SGN-U321700	8.17	6.98	10.19	11.09

Les.3519.1.S1_at	Cyclin d3-2		10.23	8.99	11.81	12.19

Les.3520.1.S1_at	Cyclin d3-2	SGN-U321308	11.06	9.68	12.2	12.64

Les.5343.1.S1_at	Cell division control protein 6	SGN-U323296	7.18	5.95	7.94	8.38

LesAffx.19390.1.S1_at	CCNB2_MEDVAG2 mitotic-specific cyclin-2 (b-like cyclin)		7.14	7.65	8.88	9.79

Replication protein a2						

Les.5740.1.S1_at	Replication protein a2	SGN-U322617	10.2	8.76	10.85	11.18

Cell cycle and cell division genes that changed in parthenocarpic fruit set with respect to normal fruit set. RP75/59 Ant_E, UC82 Ant_E, RP75/59 3DPA_E and UC82 3DPA_E columns showed the mean values of expression for each gene according to the microarray, after normalization and log2 transformation.

### Expression of genes related to hormones

Hormones play a key role in all of the development processes. Here we focused on the hormone related genes to determine which ones were involved in tomato carpel development, fruit set and to find differences between normal fruit set and parthenocarpy. We analyzed the genes regulated during normal carpel development and fruit set (variable TIME) (Additional file 6), and the genes differentially expressed in parthenocarpic fruit set (eTIME RP75/59 and eRP75/59vsUC82) (Table 5). Almost all genes that had a differential expression between parthenocarpic and normal fruit set were also differentially expressed during normal carpel development and fruit set.

**Table 5:** Hormone related genes

Array probe set	Gen description	Assigned SGN	RP75/59 Ant_E	UC82 Ant_E	RP75/59 3DPA_E	UC82 3DPA_E
ABCISIC ACID						

Les.5830.1.S1_at	Protein kinase (signaling)	SGN-U322602	9.5	9.6	9.36	8.76

LesAffx.8142.1.S1_at	Abscisic acid-induced protein	SGN-U321021	10.77	10.92	10.43	9.86

Les.4655.1.S1_at	MOSC domain protein (synthesis-degradation)	SGN-U315128	12.04	12.54	10.89	10.59

Les.112.1.S1_at	ABA1 zeaxanthin epoxidase (synthesis-degradation)	SGN-U321035	10.07	11.04	9.19	8.83

AUXIN						

LesAffx.49488.1.S1_at	Auxin-responsive family protein (signaling)		4.66	5.7	4.17	4.19

Les.3489.1.S1_at	Aminopeptidase p (transport)	SGN-U321375	10.29	10.42	9.89	9.55

Les.4501.1.S1_at	Auxin-induced protein	SGN-U313066 SGN-U334111	4.88	4.91	4.93	6.8

Les.5138.1.S1_at	Auxin-induced protein	SGN-U316711	10.55	10.38	9.43	10.43

LesAffx.2303.1.S1_at	Highlyauxin-induced protein (aldo keto reductase family)		8.09	8.32	7.41	6.61

Les.4383.1.S1_at	Metallocarboxypeptidase inhibitor (response)	SGN-U338720 SGN-U314263	13.34	11.82	13.89	10.87

Les.3707.1.A1_at	Auxin-responsive protein IAA2 (response)	SGN-U339965	4.49	4.5	4.75	5.34

Les.3707.1.S1_at	Auxin-responsive protein IAA2 (response)	SGN-U339965	4.61	4.45	5.86	8.01

Les.3702.1.S1_at	Auxin-regulated protein IAA10 (response)	SGN-U323974	5.1	4.75	5.75	4.81

Les.5154.1.S1_at	Vesicle-associated membranesynaptobrevin 7b (response)	SGN-U316860	12.18	12.46	11.76	11.62

Les.5312.1.S1_at	GH3-like protein (response)	SGN-U331710	8.35	9.65	6.83	7.39

LesAffx.67531.1.S1_at	AXR2| IAA7 (response)		4.79	4.08	6.15	8.74

ETHYLENE						

Les.354.1.S1_at	KNAT3 homeodomain protein (detection)		10.41	10.76	9.86	9.33

Les.5917.1.S1_at	ACC oxidase ACO5 (synthesis-degradation)	SGN-U323861	6.95	5.2	4.99	4.68

Les.5312.1.S1_at	GH3-like protein (ethylene-dependent resistance)	SGN-U331710	8.35	9.65	6.83	7.39

Les.5841.1.S1_at	3-keto-acyl-thiolase 2 (ethylene-dependent resistance)	SGN-U312631	10.87	11.59	10.08	10.4

Les.4233.1.S1_at	Universal stress proteinfamily protein (induced)	SGN-U315394 SGN-U315393	5.23	7.24	4.82	4.5

GIBBERELLIN						

Les.3625.1.S1_at	GASA5-like protein (induced)	SGN-U313658 SGN-U334522	9.97	10.58	9.48	11

LesAffx.9038.1.S1_at	DWARF3 (synthesis-degradation)	SGN-U334242	12.76	11.39	13.11	12.88

Les.5621.1.S1_at	GA2-oxidase (synthesis-degradation)	SGN-U325681	6.05	6.22	7.41	8.1

Les.63.1.S1_at	GA20-oxidase 3 (synthesis-degradation)	SGN-U321270	8.83	5.19	9.04	7.55

Les.65.1.S1_at	GA20-oxidase 2 (synthesis-degradation)	SGN-U333339	5.17	4.79	5.67	6.83

Hormone genes that changed in parthenocarpic fruit set with respect to normal fruit set. RP75/59 Ant_E, UC82 Ant_E, RP75/59 3DPA_E and UC82 3DPA_E columns showed the mean values of expression for each gene according to the microarray, after normalization and log2 transformation.

During carpel development and normal fruit set we detected 20 modulated gibberellin genes (Additional file 6). When we compared normal and parthenocarpic fruit set we detected 5 gibberellin related genes (Table 5). Two were GA20-oxidases, that have been verified by QPCR (Table 1). GA20-oxidase 3 was clearly activated in RP75/59 as of the flower bud stage and was not inhibited at anthesis in contrast to the UC82 pattern, whereas the other one, GA20-oxidase 2, was clearly activated at normal fruit set (UC82 3DPA) with respect to parthenocarpic fruit set. The other three differentially expressed genes were a GA2-oxidase, a GASA5-like protein and the *DWARF3 *gene (expression patterns in Table 5).

During carpel development and normal fruit set we detected 40 auxin related genes (Additional file 6). We detected 12 auxin related genes that were differentially expressed in parthenocarpic fruit set, none of which were implicated in auxin biosynthesis. One was involved in auxin transport, two in auxin signaling pathway, four were auxin induced proteins, five were related to response to auxin stimulus and one was a GH3-like protein involved in auxin and ethylene response (expression patterns in Table 5).

We also investigated the function of ethylene in ovary development and fruit set. We detected 38 ethylene related genes that were modulated during normal carpel development and fruit set (Additional file 6). Most of these (28 out of 38) showed almost the same pattern, being inactivated at 3DPA with respect to previous stages. All of the ethylene metabolism genes showed this pattern except two: s-adenosylmethionine synthetase, showed higher expression at pre-anthesis and 3DPA, and *ACS1A*, increased its expression from bud to 3DPA. There were also five genes with higher expression at flower bud and 3DPA, and three with higher expression at the flower bud to pre-anthesis stage.

When we checked the ethylene related genes differentially expressed between parthenocarpic and normal fruit set, we detected five genes (Table 5). All of these genes also changed throughout carpel development and normal fruit set. Four that were inhibited at 3DPA were more activated at the anthesis of UC82 than in RP75/59. The other gene ACO5, was verified by QPCR (Table 1). This gene is the only one related to ethylene biosynthesis was also inhibited at 3DPA; however, its expression was higher in RP75/59 with respect to UC82 in all of the analyzed stages.

We also checked the genes related to ABA and cytokinin. We found 12 ABA genes and 8 cytokinin related genes modulated during normal carpel development and fruit set (Additional file 6). When we studied the differences between normal and parthenocarpic fruit set we found four differentially expressed ABA related genes (Table 5), all of which were inhibited at 3DPA and had a bigger decrease in UC82 than in RP75/59. No cytokinin related genes were found differently expressed at parthenocarpic fruit set (Table 5).

## Discussion

Most recent studies on tomato fruit development have been focused on the ripening process [1,23-25], but only a few have included early developing fruit and fruit set [26,27]. The carpel develops before anthesis has to wait for pollination and successful fertilization signals before changing into a fruit. This relationship between pollination and fruit set can be broken to develop parthenocarpic fruit [7]. Our aim is to identify genes linked with carpel development in order to understand the transcriptional changes that will change a carpel into a fruit, and how these processes can take place in absence of pollination.

### Transcriptomic analysis of tomato carpel development and fruit set

To identify the key steps and processes in tomato carpel development and fruit set, we analyzed the carpel transcriptome at four different stages (bud, bud to preanthesis, anthesis and 3DPA) in two tomato lines (a control and a facultative parthenocarpic line). We identified 2842 modulated genes in the control line (UC82). When we clustered the modulated genes into 15 groups by their expression pattern, we observed that the differences between the two lines were mainly due to expression level, and that it was at anthesis where we found the greatest differences. These differences of expression were also detected when we clustered the experiments. Flower bud and bud to preanthesis were clustered together and then grouped with 3DPA, while all of the anthesis samples were clustered in a different group, thereby demonstrating the special nature of this stage.

With our new annotation of the GeneChip Tomato Array we analyzed the frequency of the different GO terms of the modulated genes during UC82 carpel development and fruit set with respect to the rest of the genes present in the microarray. The cell cycle genes were regulated throughout this process, as carpel cells are divide at flower bud and stop at anthesis until pollination and fertilization, which leads to fruit set when the cell division restarts [4]. We also analyzed the GO terms of the differentially expressed genes in RP75/59 (the parthenocarpic line) under normal conditions, these GO terms were the same as in the control line, and so the ability to set parthenocarpic fruits did not involve drastic changes in gene expression or the general process of carpel development. The regulated processes were the same, and the differences in gene expression between the control and the parthenocarpic line were not concentrated in any particular process.

### Differentially expressed genes in forced parthenocarpic fruit set

Our aim was to study parthenocarpic fruit set and to analyze the differences with respect to normal fruit set that could be the cause or consequence of the ability to set parthenocarpic fruits; we focused our study on the most different stage and forced parthenocarpy to improve the analysis.

When we analyzed the gene expression we detected 758 genes differentially expressed in parthenocarpic fruit set with respect to normal fruit set. The expression patterns of these genes showed that the differences between the parthenocarpic line and the control line at anthesis and 3DPA were mainly related to level expression. Moreover, the most significant differences between lines were detected at anthesis, just as in the non-emasculated samples. This suggests that parthenocarpic and normal fruit set follow similar paths and that the main differences are concentrated at the anthesis stage.

To detect the processes involved in parthenocarpic fruit set, we analyzed the GO terms of the differentially expressed genes. The GO terms were the same ones as in normal development, normal and parthenocarpic fruit set were very similar, and just a few changes confer the ability to set parthenocarpic fruits.

### Cell cycle genes during parthenocarpic carpel development and fruit set

Flower bud is a fast-growing stage in carpel development that changes at anthesis when the ovary is static, waiting for pollination and fertilization signals that will be followed by cell division during 7–10 days [4]. However, when we analyzed the cell cycle genes differentially expressed in parthenocarpic fruit set with respect to normal fruit set, most of the genes were still activated at anthesis, suggesting that cell cycle was not stopped at anthesis in the parthenocarpic line and that the carpel was already starting the first steps of fruit set. We found that genes like *CYCLIN D3:2*, that are known to be repressed at the anthesis stage in tomato and activated at 2DPA in pollinated ovaries [31,32], were actually activated at the anthesis of the parthenocarpic line.

These cell cycle activation is in concordance with previous works that reported an increase in ovary size at anthesis in other parthenocarpic lines. In the *pat *mutant at anthesis, ovaries were bigger before pollination [16]. In parthenocarpic plants carrying the *pat-2 *genes, the ovary weight was significantly higher with regard to near isogenic non-parthenocarpic lines [33].

In spite of these differences, RP75/59 fruits at 3DPA followed the same developmental paths as the UC82 ones, as all of the genes activated in the control line in response to pollination were also activated in the parthenocarpic fruit set. In contrast, parthenocarpic fruits induced by GAs application did not show this activation of all of the cell cycle related genes [27].

### Hormone related genes in parthenocarpic fruit set

The five classic hormones gibberellins, auxins, ethylene, citokinins and abcisicacid have long been known to be involved in the different developmental phases of fruits [34,6]. Here we investigated the role of those hormones in parthenocarpic fruit development.

We did not find any cytokinin related genes differentially modulated in parthenocarpic fruit set, which suggest that parthenocapy in the *pat-3/pat-4 *system might be independent of cytokinins action.

Most of the ABA related genes were activated at normal anthesis. In the parthenocarpic line, genes related to ABA showed fewer differences between anthesis and 3DPA. ABA may keep the carpel in a state of temporary dormancy at anthesis which changes to an active state upon pollination and fruit set. However, in the parthenocarpic line the anthesis is not in such state of temporal dormancy, as there were cell cycle processes active.

GAs are known to be involved in the natural parthenocarpy of tomato fruits [20]. In the GAs biosynthetic pathway there are two main points of regulation, the GA20-oxidase and the GA 3β-hydroxylase, which are subject to feedback regulation by GA action [35]. In our data, these genes followed a pattern in carpel similar to that previously described by Rebers [36] in the whole flower.*GA 3β-hydroxylase 2 *was highly expressed in anthesis, *GA20-oxidase 1 *was inhibited at anthesis, *GA20-oxidase 2 *was activated at flower bud and *GA20-oxidase 3 *was activated at 3DPA and flower bud. When we analyzed the genes related to GAs differentially expressed in the parthenocarpic line (*pat3/pat4*) with respect to the non-parthenocarpic line, we found that *GA20-oxidase 1 *was not differentially expressed and *GA20-oxidase 2 *had small differences, whereas *GA20-oxidase 3 *expression was constitutively expressed in the parthenocarpic line, even at anthesis. These results showed that the parthenocarpy in *pat3/pat4 *is mediated by an overexpression of a *GA20-oxidase 3 *in the carpel, as in the *pat *mutant where the *GA20-oxidase 1 *was constitutively expressed in the ovaries [21]. However, parthenocarpy derived from exogenous treatment with GAs leads to fruits with almost empty locular cavities [37,38], and the *pat-3/pat-4 *fruits had normal development in the locular tissue, meaning that the alteration of GAs production is not sufficient to explain this phenotype.

Auxins, such as gibberellins, are known to be involved in fruit set [39]. The application of exogenous auxins leads to parthenocarpic development with filled locules [38] like pollinated fruits. The auxin metabolism did not seem to be influenced by parthenocarpic development; however auxins were produced in response to pollination and in developing seeds [5], which stimuli were absent in these samples. Fruits from *pat-3/pat-4*, which had filled locules, can develop pseudoembryos, as has been described in *pat-2 *tomato [40], and those seed-like structures produce auxins, like the seeds in normal fruits [41].

On the other hand, there were some auxin responsive proteins whose expression was altered in the parthenocarpic line. *IAA10 *was activated only at 3DPA in the parthenocarpic line. A GH3 like protein was more activated at both stages of the non-parthenocarpic line. In addition, *IAA2 *and *AXR2|IAA7 *expression was highly activated at 3DPA with respect to anthesis, but in the parthenocarpic line this activation was clearly lower only at 3DPA in emasculated flowers. It is possible that *IAA2 *and *AXR2|IAA7 *needed pollination signals to be activated. *IAA2 *has been described as only being activated after pollination and not after GA3 treatment [27]. These different expression patterns in auxin response genes between the two lines demonstrate the complexity of auxin action in these processes and the fact that the parthenocarpic ovary was not developing exactly as would a normal one.

Ethylene plays a key role throughout fruit development and ripening in climacteric fruits and has been broadly studied [42,1,6]. In addition, ethylene has been implicated in pollination responses and in ovary development in orchid flowers [43,44]. In tomato, pollination signals and senescence will lead to an increase in ethylene synthesis followed by a decrease at 72 h after anthesis [45].

When we analyzed the expression of ethylene related genes, we found that most of the genes were inhibited at 3DPA in parthenocarpic and non-parthenocarpic carpels. This decrease in ethylene biosynthesis and signaling genes was also observed by Vriezen [27] in the ovaries of tomato flowers at 3DPA in pollinated or GA3 treated ovaries.

We investigated the differences in ethylene related genes between the carpels of the two lines. We found that *ACO5 *(1-aminocyclopropane-1-carboxylic acid oxidase 5) expression was clearly activated at anthesis in the parthenocarpic line with respect to the control in emasculated ovaries, and it was also more expressed in all of the stages tested in the parthenocarpic line. ACO (1-aminocyclopropane-1-carboxylic acid oxidase) is the last enzyme in ethylene biosynthesis and is considered to be a key point of regulation [46]. The temporal pattern of ethylene related genes and the differences between parthenocarpic and normal lines suggest a role for ethylene in carpel development and parthenocarpic fruit set. A relationship between auxins and ethylene in early stages of fruit development has been detected in the *dgt *tomato mutant, where the differential expression of subsets of the *IAA *and *ACS *genes and the alterations in fruit morphology suggest that early stages of fruit development in tomato are regulated by auxin and ethylene [47].

In *pat3/pat4 *the altered synthesis of ethylene might mimic pollination signals and may be involved in the induction of auxins synthesis and the activation of fruit set, which in normal anthesis is in a state of temporary dormancy.

## Conclusion

Transcriptomic analysis of tomato carpel development and fruit set provides a resource for future study of tomato carpel development. We identified 2842 genes regulated throughout these processes and identified the hormone related gene involved. Comparison between array experiments determined that anthesis was the most different stage and the key point at which most of the genes were modulated. We also studied the alterations of gene expression in the parthenocarpic fruit set of the *pat-3/pat-4 *system. We detected 758 genes differently modulated in parthenocarpic fruit set. These differences in gene expression were concentrated at anthesis, the key step. The most significant differences were found in cell cycle related genes. Cell cycle was not stopped at anthesis in the parthenocarpic line, contrary to normal development where carpels remain in a state of temporary dormancy, waiting for pollination signals. This dormancy state does not exist in RP75/59 anthesis is a transitional state to fruit set. We also checked the hormones related genes; GA and ethylene synthesis key genes were activated in the parthenocarpic line, and some *aux/IAA *gene expression was also altered despite the lack of differences in the auxin metabolism. In the parthenocarpic line the high expresion of GA20-oxidase 3 leads to the development of the parthenocarpic fruit ever in the absence of fertilization. Ethylene may mimic pollination signals, activating auxin synthesis and a response like that of normal fruit set. This leads to the production of pseudoembryos and fruits with normal locule development. Future work will elucidate the exact role of ethylene in fruit set and its relationship to auxin activation.

## Methods

### Plant material

Tomato lines UC82 and RP75/59, a strongly facultative parthenocarpic tomato line [15,18]; plants were grown under greenhouse conditions (24°C, 16 hours L/D).

The percentage of fruit set when the flowers were self-pollinated was greater than 90% in both lines. When the flowers were emasculated but not pollinated, the percentage of fruit set was greater than 90% in RP75/59 (all the fruits were parthenocarpic), whereas no fruit was set in the UC82 plants.

Flowers were collected at four different developmental stages and under two conditions. Time stages were flower bud (petal length between 4.5 and 7 mm), flower bud to pre-anthesis (petal length between 7.5 and 9 mm), anthesis, and 3DPA (days post anthesis). Anthesis and 3DPA flowers were collected under two different conditions, emasculated 2 days before anthesis (UC82 flowers were hand-pollinated at anthesis) and non-emasculated.

Flowers at all these stages were collected during three independent weeks to have biological replicates, the carpels were extracted, frozen in liquid nitrogen and stored at -80°C.

### RNA extraction and QPCRs

Total RNA was extracted with TRI Reagent (Sigma-Aldrich, Saint Louis, USA) following the manufacturer's instructions. The RNA was purified with the RNeasy plant mini kit (Quiagen, Hilden, Germany). For the quantitative PCR (QPCR), first strand cDNA was synthesized from 1 μg of total RNA with oligo d(T) primer, using Expand Reverse Transcriptase (Roche, Nonnenwald, Germany). We used actin (TIGR acc. TC171374) as reference, PCNA (proliferating cell nuclear antigen EMBL acc. AJ515747.1) as control of the cellular division and 10 of the differential expressed genes according to the array analysis (Primers in Additional file 7). All the samples were measured in triplicate. The level of expression was calculated normalizing using actin as reference as described by Pascual [26]. The relative levels of expression between RP 75/59 and UC-82 were log 2 transformed to make the data easily comparable with the array values.

### Microarray hybridization

For the microarray analysis we hybridized three biological replicates of RP75/59 and UC82 at each condition. Conditions were flower bud, flower bud to pre-anthesis, anthesis and 3DPA, in additon to anthesis and 3DPA emasculated 2 days before anthesis (UC82 flowers were hand-pollinated at anthesis).

cDNA synthesis and cRNA production and fragmentation for the microarray hybridization were carried out as described in the Expression Analysis Technical Manual (Affymetrix, Santa Clara, CA, USA). We employed the Affymetrix GeneChip Tomato Genome Array designed specifically for monitoring gene expression in tomato. The comprehensive array consists of over 10,000 *S. lycopersicum *probe sets to interrogate over 9,200 *S. lycopersicum *transcripts. Sequence information for this array was selected from public data sources including *Lycopersicon esculentum *UniGene Build #20 (October 3, 2004) and GenBank mRNAs up to November 5, 2004. More information can be found at the Affymetrix home page [48]. The GeneChip Arrays were hybridized, stained, washed, and screened for quality according to the manufacturer's protocol at the UCIM of the University of Valencia (Valencia, Spain).

### Data analysis

Raw data with no background subtraction were analyzed with the affy package [49] from bioconductor [50]. Raw data were transformed, background corrected by RMA, normalized by quantiles, summarized by medianpolish method and transformed into base two logarithms. Raw data and normalized data were deposited at ArrayExpress acc. number E-MEXP-1643.

Differentially expressed genes were extracted with the maSigPro package [28] from bioconductor. MaSigPro is an R package for the analysis of single and multiseries time course microarray experiments. MaSigPro follows a two step regression strategy to find genes with significant temporal expression changes and significant differences between experimental groups. The method defined a general regression model for the data. We defined a cubic regression model (degree = 3) when we analyzed four time points, and a monomial regression model when we analyzed just two time points. First, we adjusted this global model by the least-squared technique to identify differentially expressed genes and selected significant genes by applying a false discovery rate (Q = 0.01). Secondly, a variable selection procedure was applied to find significant variables for each gene, for which we employed a stepwise regression (step.method = "two.ways.backward", alfa = 0.01). Then, lists of differentially expressed genes according to each variable were generated (rsq = 0.6). After the maSigPro analysis, a difference of 0.75 at the logarithmic scale (fold-change greater than 1.68) was required to consider a gene differentially expressed.

To cluster the samples, we discarded the constant genes in order to avoid background noise. We made a hierarchical cluster with Euclidean distance by UPGMA method (bootstrap 100 replicates).

To create the gene clusters, we employed the K-means method [51] with Pearson correlation distances.

### Microarray annotation and functional analysis

The GeneChip Tomato Genome Array was re-annotated using the Blast2GO package [29], which assigns the GO terms based on the BLAST definitions. The GeneChip Tomato Genome Array probe sequences were downloaded from the Affymertix home page [48]. A blastx was made against the NCBI nr-database of 2007-09-11, and a E-value < 10-10 level was required to take into account the blast result. The Blast2GO improved the annotation by comparing our sequences against the InterPro domains database using InterProScan [52] at the EBI server. Finally each probe was annotated to the higher GO level possible according to the information extracted from the blastx and the InterProScan analysis. Our GO term annotations were used in the fatiGO [30] for the functional analysis. To improve the annotation of the selected genes, we also made a blastn against the SGN tomato unigenes to detect the SGN unigene represented by each array probe.

## Authors' contributions

LP obtained the experimental data and participated in the microarray analysis. JC designed the study and experiments and participated in the microarray analysis. JMB did the bioinformatic analysis to obtain the microarray annotation. LP and FN prepared the manuscript. All authors read and approved the final manuscript.

## Supplementary Material

Additional file 1 **TIME variable genes**. Differentially expressed genes throughout normal carpel development and fruit set. Click here for file

Additional file 2 **TIME RP75/59 and UC-82vsRP75/59 variables genes**. Differentially expressed genes throughout carpel development and fruit set in the parthenocarpic line with respect to the non parthenocarpic one. Click here for file

Additional file 3 **Tentative annotation of Affymetrix tomato GeneChip**. Array probe set were annotated to the higher GO level possible in each case. Click here for file

Additional file 4 **eTIME RP75/59 and eUC-82vsRP75/59 variables genes**. Differentially expressed genes in parthenocarpic fruit set with respect to the non-parthenocarpic one. Click here for file

Additional file 5 **Cell division and cycle genes**. Cell cycle and cell division genes that changed during normal carpel development and fruit set (TIME). Click here for file

Additional file 6 **TIME variable genes related to hormones**. Hormone related genes modulated along normal carpel development and fruit set. Click here for file

Additional file 7 **QPCR primers**. Primers employed in the microarray verification. Click here for file
